# On the Anatomy and Diseases of the Urinary and Sexual Organs, Containing the Anatomy of the Bladder and Urethra, and the Treatment of the Obstructions to Which These Passages Are Liable

**Published:** 1845-07

**Authors:** 


### BIBLIOGRAPHICAL NOTICES.

*On the Anatomy and Diseases of the Urinary and Sexual Organs, containing the Anatomy of the Bladder and Urethra, and the Treatment of the Obstructions to which these passages are liable.* By G. J. Guthrie, F. R. S., &c. From the third London edition; pp. 150, 8vo. Philadelphia, Lea & Blanchard, 1845. (From the Publishers.)

We propose, in a brief analysis, to give our readers a view of the contents of this volume, dwelling particularly upon such parts as are of peculiar interest, from their novelty or importance.

Chap. I, embracing 26 pages, treats of the anatomy of the bladder. In addition to a pretty full and clear description of this organ, as usually given, the author advances the opinion, that the peculiar structure about the mouths of the ureters, is designed to keep them always patulous, except when the bladder is distended, when they are pressed upon and closed, in order to delay if not to prevent the flow of urine into the kidney. The effect of this is to check the secretion, diminishing it from over to less than 1 pint in twenty-four hours, by retaining it in the ureters, and thus pressing upon the kidney. Another peculiar view of our author, is that the little projection, below the orifice of the bladder, called by Sir Everard Home, the third lobe of the prostate, and by the French, *luette vesicale*, is in fact only a part of the coats of the bladder, and is not a part of the prostate.

Chap II, of 22 pages, is devoted to the structure of the urethra. Contrary to the received opinions, our author regards this as not muscular, but as a compensation, he allows it to be possessed of a peculiar contractility, which would reduce the controversy to one of words rather than of structure. We do not, however perceive, that he has advanced anything to induce a change of views on this part of the question.

Chap. III embraces 13 pages on the formation of spasmodic and permanent strictures. In reference to the former, they are thought by Mr. Guthrie to be very rare, and those so called he considers as engorgements of the mucous membrane, best relieved, he says, by the immediate introduction of a catheter.

The chapters on the treatment of permanent and impassable stricture, are very full and judicious, surpassing very much in merit, those devoted to the anatomical descriptions. The method in almost universal use, for the cure of permanent stricture of the urethra, is by dilatation with gum-elastic bougies. We say almost, for Ricord persists in treating the great majority of these by lunar caustic; and notwithstanding theoretical views, or general opinion, our own observations bear witness to the safety and success of his treatment. The caustic, well applied, acts by removing inflammation of the passage, and the relief which it affords, proves the great agency which this state has in aggravating the disease. Mr. Guthrie does full justice to this method, which, he remarks, has fallen into unmerited neglect. He, however, in most cases, prefers to use dilatation, either from the beginning, or after having allayed irritation by caustic. He prefers the wax bougies for exploration, and those of gum-elastic, or of silver, for removing the stricture. When this is near the orifice, he practices division with the knife. We have found rupture by passing a sound through it at once, to answer perfectly.

When the stricture has become impassable, there are two methods which may be used: 1st, pass a bougie down, so as to press its extremity upon the hardened gristly substance of which it is formed; this repeated for several days, will frequently produce absorption, and allow the instrument to pass. When this is not the case, he recommends passing a catheter down to the stricture, making an inicisoin from the perineum into the urethra, behind it, so as to discharge the water, dividing the stricture, and passing a catheter into the bladder, allow this to remain till the wound heals. This, or some operation, which like it, discharges the urine and divides the stricture, is the one generally approved at the present time.

It will be seen that our author is not exclusive in his mode of treatment, and the cases in which the different methods are to be chosen, are very accurately discriminated.

There is a chapter on suffusion and retention of urine, which we pass over, in order to make room for some remarks on a subject less understood, to which the last chapter is devoted, viz.: irritation of the membranous and prostatic parts of the urethra.

These, the author ascribes, in many cases, to the irritating qualities of the urine, and recommends a careful examination of this fluid, in reference to its more prominent chemical properties. The treatment is to be regulated by the result. In other cases they are dependent upon an affection of the spinal cord, which renders the patient unable to evacuate all the urine; in others, upon gonorrhea, upon piles or other disease of the rectum, &c. In nearly all cases, when other remedies fail, opium allays the pain, and affords great relief. It is obvious, that in all these different classes of cases, regard must be had to the cause; but in removing the irritation, we have found nothing so effectual as the cauterization with the Nit. Arg., as recommended by Lallemand, which, however, Mr. Guthrie does not recommend. The name of the author, and his position as surgeon of the Westminister Hospital, will sufficiently commend the work. We can add, that from careful perusal, we have found it a most useful source of information, in relation to the diseases of which it treats. D. B.

